# Adaptive Cluster Synchronization of Directed Complex Networks with Time Delays

**DOI:** 10.1371/journal.pone.0095505

**Published:** 2014-04-24

**Authors:** Heng Liu, Xingyuan Wang, Guozhen Tan

**Affiliations:** Faculty of Electronic Information and Electrical Engineering, Dalian University of Technology, Dalian, China; Universiteit Gent, Belgium

## Abstract

This paper studied the cluster synchronization of directed complex networks with time delays. It is different from undirected networks, the coupling configuration matrix of directed networks cannot be assumed as symmetric or irreducible. In order to achieve cluster synchronization, this paper uses an adaptive controller on each node and an adaptive feedback strategy on the nodes which in-degree is zero. Numerical example is provided to show the effectiveness of main theory. This method is also effective when the number of clusters is unknown. Thus, it can be used in the community recognizing of directed complex networks.

## Introduction

During last decade, the study of complex networks has become a hot topic in various fields like physics, mathematics, biology, social sciences, computer sciences, and so on [1]–[3]. Most of complex networks have two properties: small-world and scale-free [4]–[5]. Recently, as one of the most important phenomenon of dynamical system, synchronization has gained growing attention. So far, many different kinds of synchronization in complete networks are realized, such as generalized synchronization, phase synchronization, cluster synchronization and so on [6]–[25]. Nowadays, cluster synchronization has been widely and thoroughly studied because it can show the community of the complex networks [26]–[30].

Cluster synchronization is a middle state of the progress which is from none-synchronization to complete synchronization. When this middle state is achieved, the nodes in same group (or community, or cluster) can achieve complete synchronization, but the nodes in different clusters are chaotic. Owing to the significant application in biological science and communication engineering, the researching of cluster synchronization is focus on the control method such as pinning control, adaptive control, impulsive control, and so on, but few of them studies cluster synchronization of directed complex networks with time delays.

Liu and others researched generalized synchronization of three typical complex dynamical networks including scale-free network, small-world network, and a family of interpolating network [7]. They found that there is a general progress to global generalized synchronization (GS): non-GS → partial GS → global GS and the GS stats from a small part of hub nodes with larger degrees first. In their paper, the partial GS is called cluster synchronization. Several interesting adaptive and impulsive synchronization criteria are attained for a general complex dynamical network with two different clusters by Shi and others in [8]. Lu proposed a novel adaptive strategy to make a network achieve cluster synchronization in [9], and Liu and others investigated the cluster synchronization with intermittent control in [10]. They also pointed out that to realize cluster synchronization, enlarging the couplings of nodes in the same cluster is the key point.

There are also some papers on cluster synchronization of directed networks without time delays. Ma and others intensively studied the pinning cluster synchronization of directed complex networks in [11]. They gave the pinning controllers which are applied to inter-act nodes and intra-act nodes with zero in-degree, respectively.

This paper uses an adaptive controller to make a directed network with time delays achieved cluster synchronization. The rest part of the paper is shown as following. In Section 2, the model of directed complex dynamical network and some preliminaries are given. The main theorems and corollaries for cluster synchronization through adaptive control are given in Section 3. At last, a numerical simulation is provided to show the effectiveness of the theoretical results. Conclusions are finally drawn in Section 5.

## Preliminaries

Consider a directed complex network with *N* identical coupled nodes:
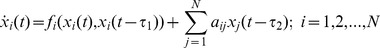
(1)Here 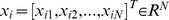 is the state vector of node *i*; function 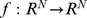 is a nonlinear function which can describe each node's dynamics; 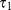 and 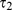 are time-varying delay and coupling delay, respectively. Matrix 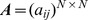 represents the topological structure of the network. In a directed complex network, 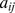 is defined as follows: if there is a direct link from node *i* to node 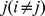, then 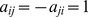; otherwise 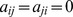. Matrix ***A*** is satisfied with diffusive condition as follows:
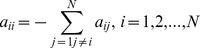
Because the complex network is directed, this paper doesn't assume ***A*** as symmetric or irreducible like other papers. The in-degree of node *i* is defined as:
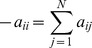
If the *i*th node is satisfied with the following equation
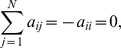
this paper will call the node which is under this condition as 0-in-degree node.

Assume that the network has *P* clusters (*P* is unknown), which means that all nodes in network will split into *P* groups when the network achieves cluster synchronization. If 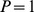, then cluster synchronization turns to complete synchronization. If node 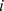 belongs to the *k*th cluster, this paper denotes that 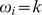. When the complex network achieves cluster synchronization, for any node 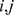, the following equation is established.
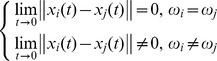
That is to say, when the network achieves cluster synchronization, the community of the network can be recognized. If it defines a solution vector 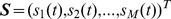 to represent the desired state when the network achieves cluster synchronization at time *t*, here 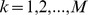. The error system is defined as follows:
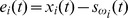
Here the stable dynamic status 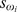 is satisfied with 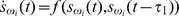. The complex network can be considered to achieve P-cluster synchronization when the following condition is satisfied:
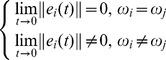
(2)


## Adaptive Cluster Synchronization

In order to make complex network Eq. (1) achieved cluster synchronization, an adaptive controller 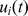 is added on each node. The controlled dynamic network can be rewritten as
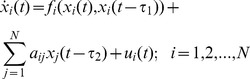
(3)The controller is designed as following:
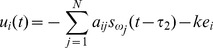
(4)Here, the constant 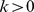. The error system of Eq. (2) can be obtained as
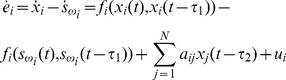
(5)Throughout this paper, the following assumptions are needed to prove the main theorem.

### 

#### Assumption 1

If there is a nonlinear dynamical function 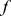, to any state vectors 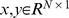, there exists a constant 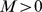 to make the following equation established:
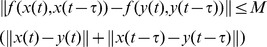
(6)


#### Remark 1

Assumption 1 holds as long as 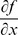 are uniformly bounded. Almost all well-known dynamical chaotic and hyper chaotic systems have the form of Eq. (3), which meets the condition of assumption 1 [31].

#### Assumption 2

There exists a constant 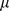 which can make a differentiable time-varying delay 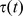 satisfied the following equation.
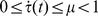
It is clearly that assumption 2 is valid for constant 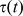.

1. When the network has no 0-in-degree node.

#### Theorem 1

Under assumption 1 and 2, the controlled complex network Eq. (3) with adaptive controller Eq. (4) can achieve cluster synchronization if *k* is satisfied with the following equation
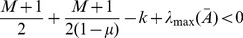
(7)Here, *M* and 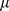 are positive constants, and
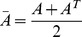



#### Proof

Define a Lyapunov function:
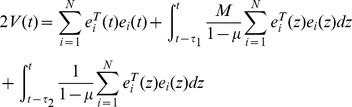
(8)Calculating the time derivative of 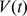 along the trajectories of Eq. (4), one has
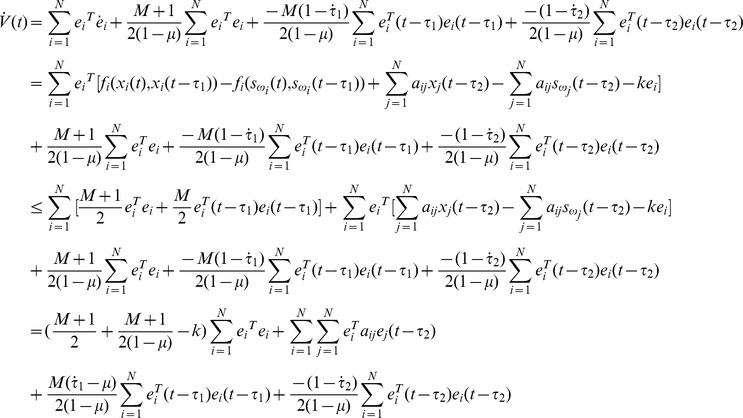
If the equation 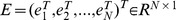 is denoted, one gets
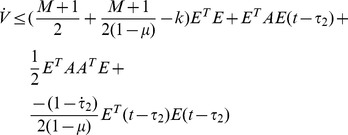
According assumption 1, one gets
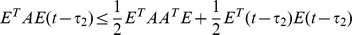
Thus, under assumption 2 and Eq. (7), the following equation can be established.
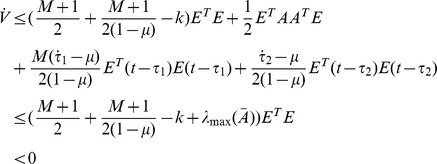
Thus Eq. (2) can be satisfied by the condition as Eq. (7), the proof is completed.

2. When the network has some 0-in-degree nodes.

0-in-degree nodes just send information into network but do not receive information from other nodes. That is to say, 0-in-degree nodes are hard to achieve synchronization. This can be proved as following. The linearization of Eq. (5) with controller Eq. (4) can be rewritten as
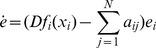
(9)Here, 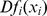 is the Jacobi matrix of function 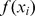 at 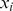. If the *i*th node is 0-in-degree node, one has
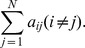
So 0-in-degree node has
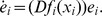
Since 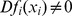, 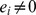, the error system of 0-in-degree node is hardly to equal 0. It means that the network is hardly to achieve synchronization.

In order to make the network which has 0-in-degree nodes achieved cluster synchronization, this paper designs a feedback adaptive strategy on 0-in-degree nodes.

#### Theorem 2

Under assumption 1 and 2, if complex network Eq. (3) has 0-in-degree nodes, it can achieve the desired cluster synchronization if controller Eq. (4) and adaptive condition Eq. (7) hold, and adaptive feedback strategy is given as Eq. (10), and Eq. (11) is established.
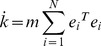
(10)

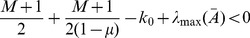
(11)where 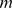 is a positive constant, 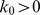 is a known constant.

#### Proof

Define a Lyapunov function as
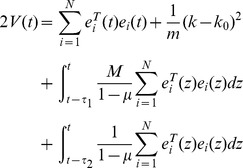
Calculating the time derivative of 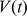 along the trajectories of Eq. (4), one has
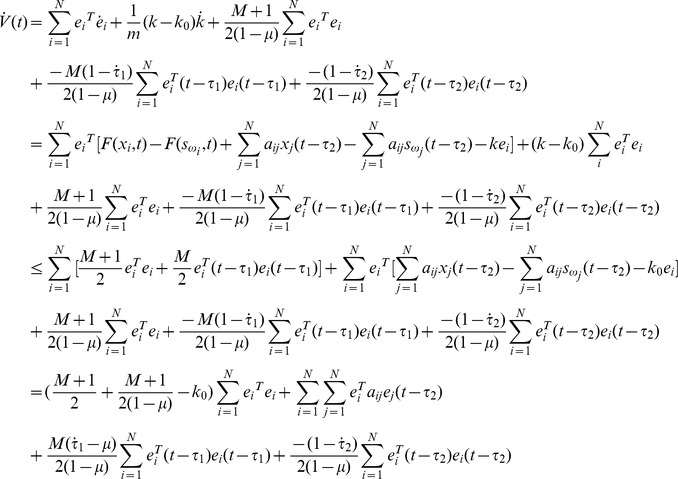
According the proof of theorem 1, under assumption 1 and 2, one gets 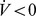 if the following equation is established.
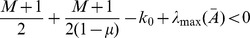
The proof is complete.

## Simulation

This section will give some examples to verify the effectiveness of the proposed theorems in section 3. In the following numerical simulations, 3-dimensonal Lorenz system is designed as the dynamical of each node. Lorenz function can be described as following:
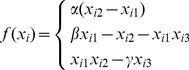
(12)When the parameters are chosen as 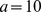, 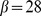, 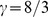, Lorenz system is chaotic. Under these parameters, the nodes' dynamics can be described as
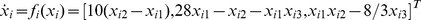
(13)


### Example 1

**Figure 1:**
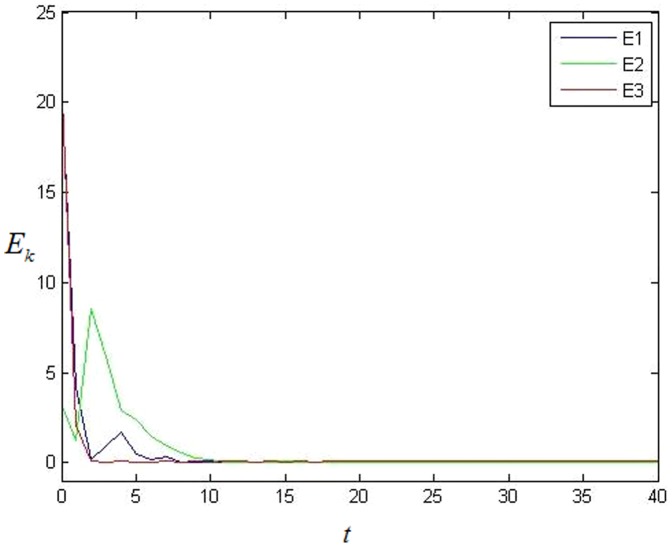
The average value of error system in BA scale-free network without 0 in-degree nodes.

**Figure 2:**
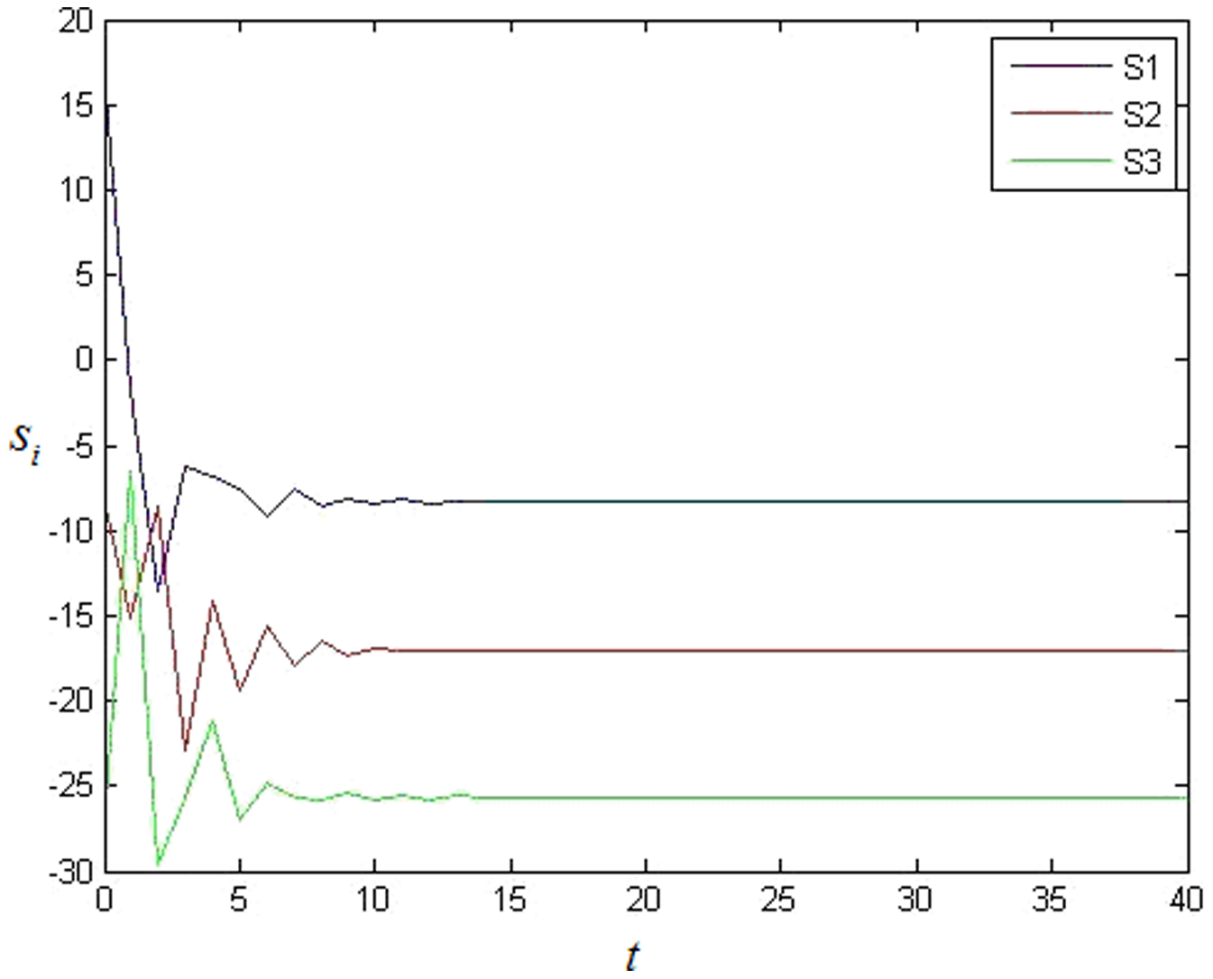
The value of each cluster's stable state in BA scale-free network without 0 in-degree nodes.

In this simulation, a directed BA scale-free network is constructed. The detail generation algorithm for BA scale-free network is introduced in [5]. The parameter are 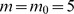, 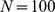. Because the network is directed, when the *i*th node and the *j*th node are connected from node *i* to node *j*, then 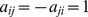. Each node of the network is controlled as Eq. (4), and the in-degree of each node is not 0, which means the following equation will be established for each node:
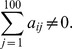



The attractor of Lorenz system is bounded by 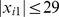, 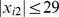, 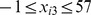, 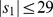, 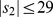, 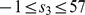, thus the network has three clusters. In the following simulation, this paper will use the method in section 3 to confirm the number of clusters is three. According to theorem 1, the dynamic network Eq. (2) with controller Eq. (4) can achieve cluster synchronization when Eq. (7) is established. In order to measure the quality of the process of cluster synchronization, this paper uses the following quantities:
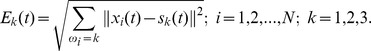
Here 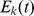 represents the average of each error system. When the network achieves cluster synchronization, the following equation will be satisfied:
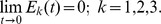



The simulation result is shown in the following. It's easy to see that each error system is 0 at last which means that each cluster achieves synchronization from Fig. 1. Fig. 2 shows the value of 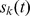. Because 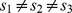 when 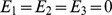, it is shown that the nodes in different cluster cannot achieve synchronization clearly. The network has three clusters according to the simulation result.

### Example 2

In this example, this paper uses a directed WS small-world network. The number of nodes as the BA scale-free in example 1. In detail, this paper will use the parameter to construct a WS small-world network as [4], the rewiring probability is 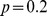, the number of nodes is 100, and 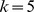. In this example, there are some nodes will be chosen randomly as 0-in-degree nodes.

**Figure 3:**
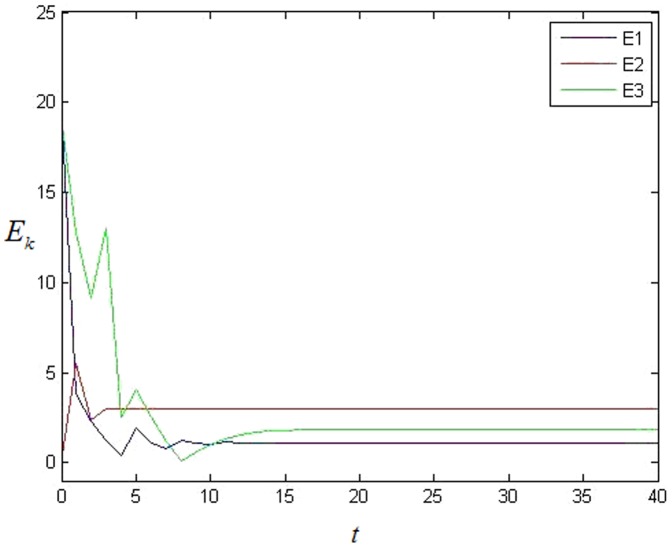
The average value of error system without adaptive feedback in NW small-world network with 0 in-degree nodes.

**Figure 4:**
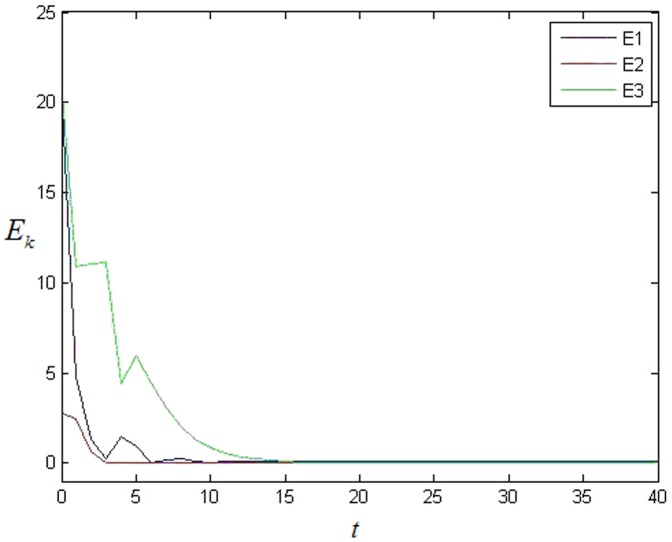
The average value of error system with adaptive feedback in NW small-world network with 0 in-degree nodes.

**Figure 5:**
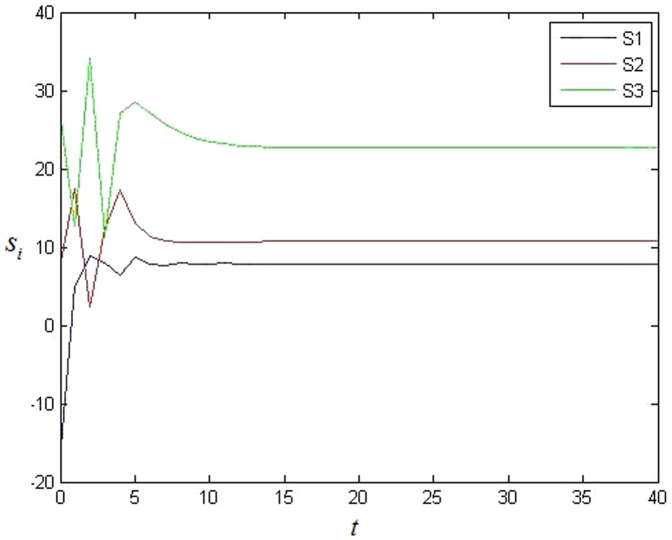
The value of each cluster's stable state with adaptive feedback in NW small-world network with 0 in-degree nodes.

At first, controller Eq. (4) is added on each node. The simulation result is shown as Fig. 3. It is easy to see that each cluster can't achieve synchronization at all because each error system cannot achieve 0. Then the adaptive feedback strategy Eq. (10) is added on each 0-in-degree node, here. The simulation result shows that the network can achieve cluster synchronization as Fig. 4 and Fig. 5. It is easy to see that the network can achieve cluster synchronization when the adaptive feedback strategy as Eq. (10) is used. It is easy to see that the number of clusters is three in Fig. 5.

## Conclusion

In this paper, cluster synchronization of directed complex dynamic network with time delays was investigated. An adaptive controller is added on each node and feedback strategy is added on 0-in-degree nodes. When the cluster synchronization is achieved, the community of the network also can be recognized. The numerical simulation has demonstrated the effectiveness of the proposed approach. First, a BA scale-free network without 0-in-degree node was investigated. The number of the clusters is unknown. After adding an adaptive controller in theorem 1 on each node, the network can achieve cluster synchronization, and the community of the network also can be recognized correctly. Then, a WS small-world network with some 0-in-degree nodes was investigated. The simulation result showed that only adding the controller in theorem 1 cannot make network achieved cluster synchronization. But if using the adaptive feedback controller in theorem 2, the network can achieve cluster synchronization, and the community of the network can be recognized.
